# Epidemiology of autonomic dysfunction in Parkinson's disease (Review)

**DOI:** 10.3892/mi.2025.267

**Published:** 2025-09-01

**Authors:** Jamir Pitton Rissardo, Ahmed Farid Gadelmawla, Ibrahim Khalil, Ayah Abdulgadir, Karandeep Singh Bhatti, Ana Letícia Fornari Caprara

**Affiliations:** 1 Department of Neurology, Cooper University Hospital, Camden, NJ 08103, USA; 2 Faculty of Medicine, Menoufia University, Menoufia 6132720, Egypt; 3 Faculty of Medicine, Alexandria University, Alexandria 5372066, Egypt; 4 Faculty of Medicine, University of Khartoum, Khartoum 11115, Sudan

**Keywords:** dysautonomia, autonomic insufficiency, autonomic dysfunction, autonomic nervous system, premotor phase, predictive biomarker, orthostatic hypotension, gastrointestinal, cardiovascular, sialorrhea

## Abstract

Although autonomic dysfunction symptoms are commonly reported by patients with Parkinson's disease (PD) (70-90%), they are frequently under-recognized. Dysautonomia often precedes motor symptoms and can affect the quality of life (QoL) of patients with PD. The present review provides a summary of evidence on prevalence patterns, risk factors and clinical presentations from organ systems related to autonomic dysfunction. Cardiovascular symptoms include orthostatic hypotension (30-50%), supine hypertension (34-50%) and non-dipping patterns of blood pressure (83-88%). Constipation is commonly observed during the prodromal period (60%), and is observed in up to 100% of patients with PD. Genitourinary (89%) and sexual dysfunctions (52-75%) are common, although under-reported. An older age, male sex, duration of disease, severity of the disease and akinetic-rigid phenotype are directly related to overall worse dysautonomia. Genotypic variants have varying degrees of relation with autonomic symptoms; for example, the SNCA gene mutation is associated with cardiac sympathetic denervation, and PARK2 or PARK9 are related to mild effect in autonomic function. Autonomic symptoms are associated with more rapid progression of disease, the attainment of disease milestones, cognitive decline and a poorer QoL. The true prevalence of dysautonomia may be higher due to of the variability of presentation and reporting biases, and current diagnostic definitions may underestimate these non-motor symptoms. The early detection of autonomic impairment may provide time points for intervention that could modify the natural history of the disease. Future studies are required to be directed towards PD-related treatment strategies, autonomic-cognitive relationships, and the development of better animal models covering the complex pathophysiology of PD.

## 1. Introduction

**Figure 1:**
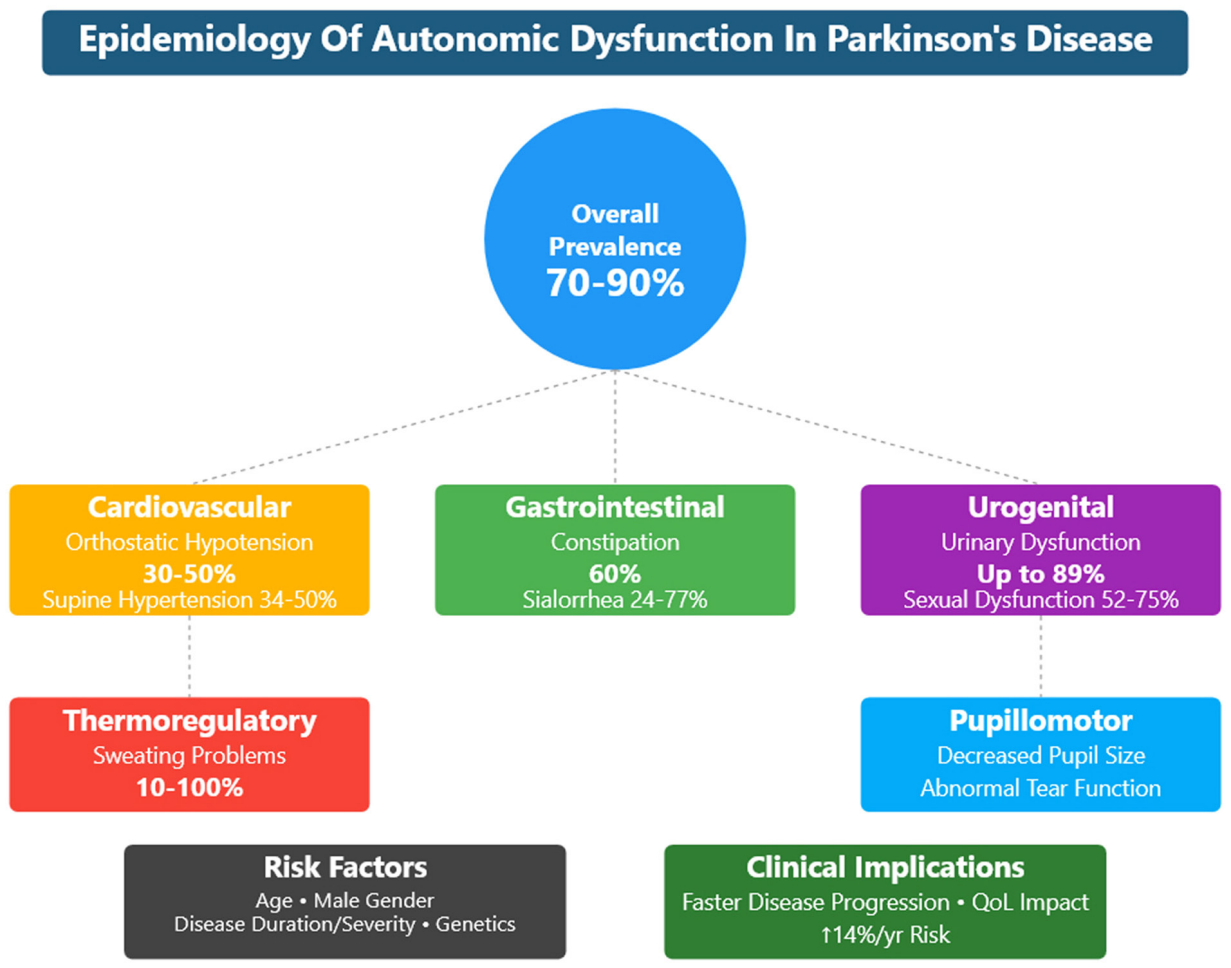
Epidemiology of autonomic dysfunction in Parkinson's disease.

Parkinson's disease (PD) is a progressive neurodegenerative disorder that is recognized mainly for presenting clinically with motor symptoms of tremors, rigidity and bradykinesia (Fig. 1) (1). However, non-motor symptoms are often underrecognized in PD, particularly those related to autonomic functions (2). Autonomic dysfunction can affect a wide array of systems, including cardiovascular (3), gastrointestinal (4), genitourinary (5), sweat glands (6), sexual function (7) and even temperature regulation (8).

**Figure 2:**
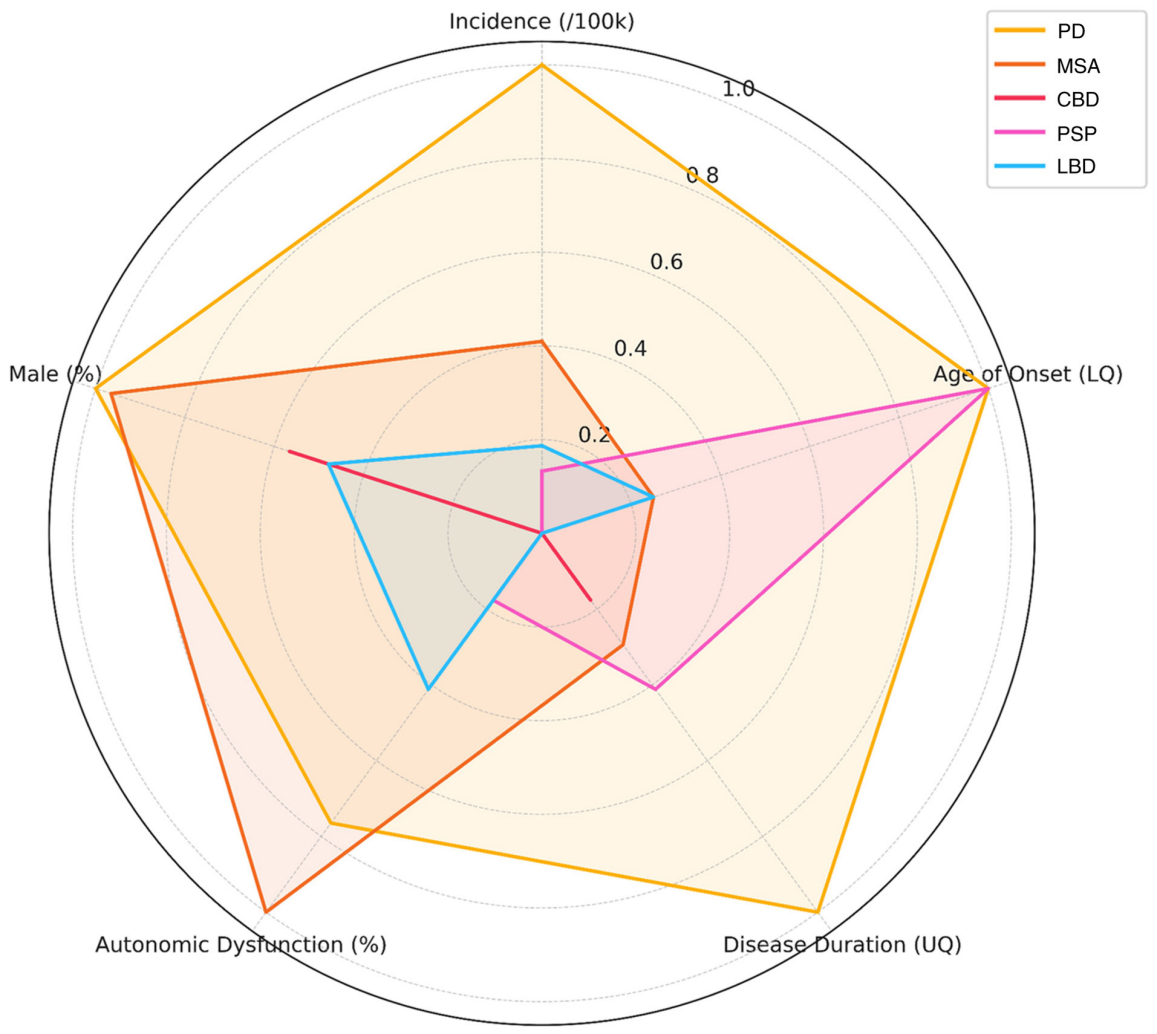
Radar chart of the epidemiological data of autonomic dysfunction in Parkinson's disease and atypical parkinsonism. The variables analyzed were incidence of the disease, male percentage of patients, percentage of autonomic dysfunction, upper quartile of the disease duration, and lower quartile of the disease onset. CBD, corticobasal degeneration; LBD, Lewy body dementia; LQ, lower quartile (25%); MSA, multiple system atrophy; PD, Parkinson's disease; PSP, progressive supranuclear palsy; UQ, upper quartile (75%). Consider reading supplementary material (Table SII) for specific information regarding percentages (14,15).

While less visible than the motor symptoms, autonomic dysfunction significantly affects the quality of life (QoL) of patients with PD (9). It can manifest early in the course of the disease, sometimes even preceding the onset of motor symptoms (10), and its prevalence increases with disease progression (11,12). In this context, orthostatic hypotension (OH) is related to significant increases in the health care burden from $9831 to $25,205/person/year (13). Notably, PD and atypical parkinsonian syndromes are affected differently by autonomic symptoms (Fig. 2, and Tables SI and SII) (14-16).

Patients with autonomic dysfunction may have a distinct phenotype. Research has demonstrated that dysautonomia mainly manifests in older individuals with bilateral onset and lower frequency of dystonic symptoms (17). On the other hand, there are some contradictory results in the literature reporting patients with dysautonomia and worsening motor and cognitive outcomes (18). Of note, as demonstrated in a previous study, three main clusters of patients with autonomic dysfunction have been found: Cluster F (hypertension and sexual dysfunction), cluster G (depression and weight loss), and cluster H (sweating and anxiety) (19). The authors of that study did not find a specific constellation of manifestations for urinary and gastrointestinal symptoms (19). Another study found two groups of individuals defined by cluster A (thermoregulatory, gastrointestinal, and pupillomotor) and cluster B (cardiovascular and gastrointestinal). Only cluster B was associated with cognitive impairment (20). Some authors have hypothesized that non-motor symptoms, including autonomic symptoms, also fluctuate and are dependent on the levodopa equivalent daily dose (LEDD) (21).

Stanković *et al* (22) reported that autonomic dysfunction is common at the time of diagnosis of PD and progresses independently from motor symptoms during the first 3 years. De Pablo-Fernandez *et al* (23), reported a shorter survival rate with early autonomic dysfunction symptoms, a similar pattern of survival rate and autonomic dysfunction was found in patients with PD dementia (24). Moreover, patients with drug-induced parkinsonism that develop autonomic failure features more likely develop PD (25).

Understanding the autonomic dysfunction spectrum in PD is essential for clinicians and researchers. The appreciation of its early presence may provide a critical window for intervention with disease-modifying therapy, which could affect the eventual course of the disease (26). In addition, studies on autonomic dysfunction, particularly studies on individuals with idiopathic REM sleep behavior disorder (RBD), have contributed to the understanding of the pathophysiology of neurodegenerative synucleinopathies, as certain autonomic symptoms are associated with an increased risk of phenoconversion to PD or Lewy body dementia (LBD) (27). Notably, the female sex and gastrointestinal dysfunctions have been shown to be associated with phenoconversion (28,29). Furthermore, the presence of autonomic dysfunction in patients with idiopathic RBD is associated with a higher phenoconversion rate, although it is not commonly studied in clinical trials (30,31).

Recent results from the Parkinson's Progression Markers Initiative (PPMI) revealed no association between biomarkers and autonomic dysfunction. Thus, the diagnosis is still based on clinical evaluation (32). There are several challenges in diagnosing and instituting therapy for autonomic dysfunction. The symptoms are frequently non-specific and diverse, mimicking other conditions, such as adverse effects from medications (33). Moreover, several patients with PD may be hesitant to report such autonomic symptoms, fearing the loss of independence or underestimating their impact on the QoL. It is worth mentioning that autonomic dysfunction and the presence of at least one of the 44 most common single-nucleotide polymorphisms which are linked to PD can predict the diagnosis of PD in 82% of cases (34).

The present literature review on the epidemiology of autonomic dysfunction in PD is crucial due to the significant impact of these often-overlooked symptoms on the lives of individuals living with PD (9). Therefore, proactive screening and thorough assessment are paramount for the detection and management of autonomic dysfunction in PD. In recognition of these diverse presentations of this commonly hidden burden, clinicians can provide more holistic care for patients with PD, enhancing the QoL and probably altering the course of the disease.

## 2. General prevalence and incidence of autonomic dysfunction in Parkinson's disease

Despite its significant impact, the true prevalence of dysautonomia in PD remains unclear. Existing studies report a wide range of estimates, depending on the specific autonomic function being investigated and the methods used to assess it (35). This variability underscores the need to synthesize existing knowledge and identify potential gaps in the understanding of how frequently different types of autonomic dysfunction occur in people with PD.

Autonomic dysfunction is common among patients with PD, affecting up to 70-90% of patients (36,37). The dysfunctions in the cardiovascular system manifests as orthostatic hypotension (OH) (38). OH is a sustained fall in blood pressure (BP) ≥20 mmHg systolic or ≥10 mmHg diastolic when moving from the supine to the standing position. OH is a clinical sign, and it can be symptomatic or asymptomatic. When symptomatic, it manifests as lightheadedness, syncope (39), blurry vision and feeling faint, which can be explained by tissue hypoperfusion. Other less specific symptoms include tiredness, cognitive impairment, dyspnea, neck and shoulder discomfort, or angina (38). The prevalence of OH in PD is 30-50%, although it is symptomatic in approximately one-third of patients (13-16%). The prevalence increases with age and disease progression (23). Supine hypertension (SH) is also common among patients with PD. The American Heart Association defined three degrees of arterial hypertension in the case of BP as ≥140 mmHg systolic or ≥90 mmHg diastolic (40). The prevalence of SH was found in 34-50% of patients with PD (41,42). OH can cause acute morbidities, such as syncope and falls, whereas SH causes end-target organ damage over time (38). SH and OH have been proposed as negative prognostic factors for survival, cardiological and cerebrovascular outcomes, as well as cognitive decline in PD (43).

The gastrointestinal system is also affected by autonomic dysfunction, with a number of clinical manifestations. Upper and lower gastrointestinal symptoms, such as constipation, are common in patients with PD and contribute to a decreased QoL (44). At least 35% of patients with PD complain of dysphagia (45). As shown by videofluoroscopy, the majority of patients with PD have abnormal swallowing with reduced efficiency and frequency. This manifests as sialorrhea or the drooling of saliva in 50-60% of patients with PD, particularly those with advanced disease (46). Constipation is the most common autonomic and gastrointestinal symptom and occurs in up to 90% of patients with PD (47). Autonomic dysfunction of the gastrointestinal system may present as acute emergencies, such as colonic volvulus, intestinal pseudo-obstruction, megacolon, fecal impaction, or overflow diarrhea (48).

Urinary dysfunction often affects patients with PD due to the high prevalence of detrusor overactivity. Up to 85% of patients with PD suffer from lower urinary tract symptoms (49). Nocturia is the most commonly reported symptom (57-86%), followed by frequency (32-71%), urgency (32-68%) and urge incontinence (21-40%). Hesitancy and incomplete emptying affect 1-38% and 8-28% of patients with PD, respectively (50-52). Sexual dysfunction is also common among patients with PD and can appear years prior to the diagnosis of PD (53). Of note, 79% of males with PD complain of erectile dysfunction, ejaculation issues and difficulties achieving orgasm. In addition, 75% of females with PD and multiple system atrophy (MSA) report sexual-related issues, such as as vaginal dryness, decreased libido and difficulties reaching orgasm (38).

## 3. Demographics and risk factors

*Age.* Age is associated with autonomic dysfunction and has direct and indirect associations with this dysfunction. Age has been found to be related to autonomic dysfunction, with older patients having more autonomic dysfunction symptoms (23). Szewczyk-Krolikowski *et al* (54) found only a trend without statistical significance between age and autonomic dysfunction, whereas Zhou *et al* (55) revealed that age was an independent predictor of dysautonomia. Furthermore, older patients with PD are most likely to have more severe disease with a longer disease duration. Autonomic dysfunction has been found to be prevalent in patients with more severe motor disease (47,56).

*Female/male sex.* The male sex is more commonly associated with autonomic dysfunction (23), and males have more drastic impairment of the autonomic functions (57). In this context, males are more likely to be treated for autonomic dysfunction (58). One of the possible explanations for this finding is that physicians screen for symptoms based on the sex of patients (59). It has been found that urinary and sexual symptoms are more common in males with PD, while cardiovascular dysfunction is more common among females (59,60). However, some researchers have found that the prevalence of dysautonomia is more common among females, which may be explained by regional and cultural factors of where the study was conducted (61).

**Figure 3:**
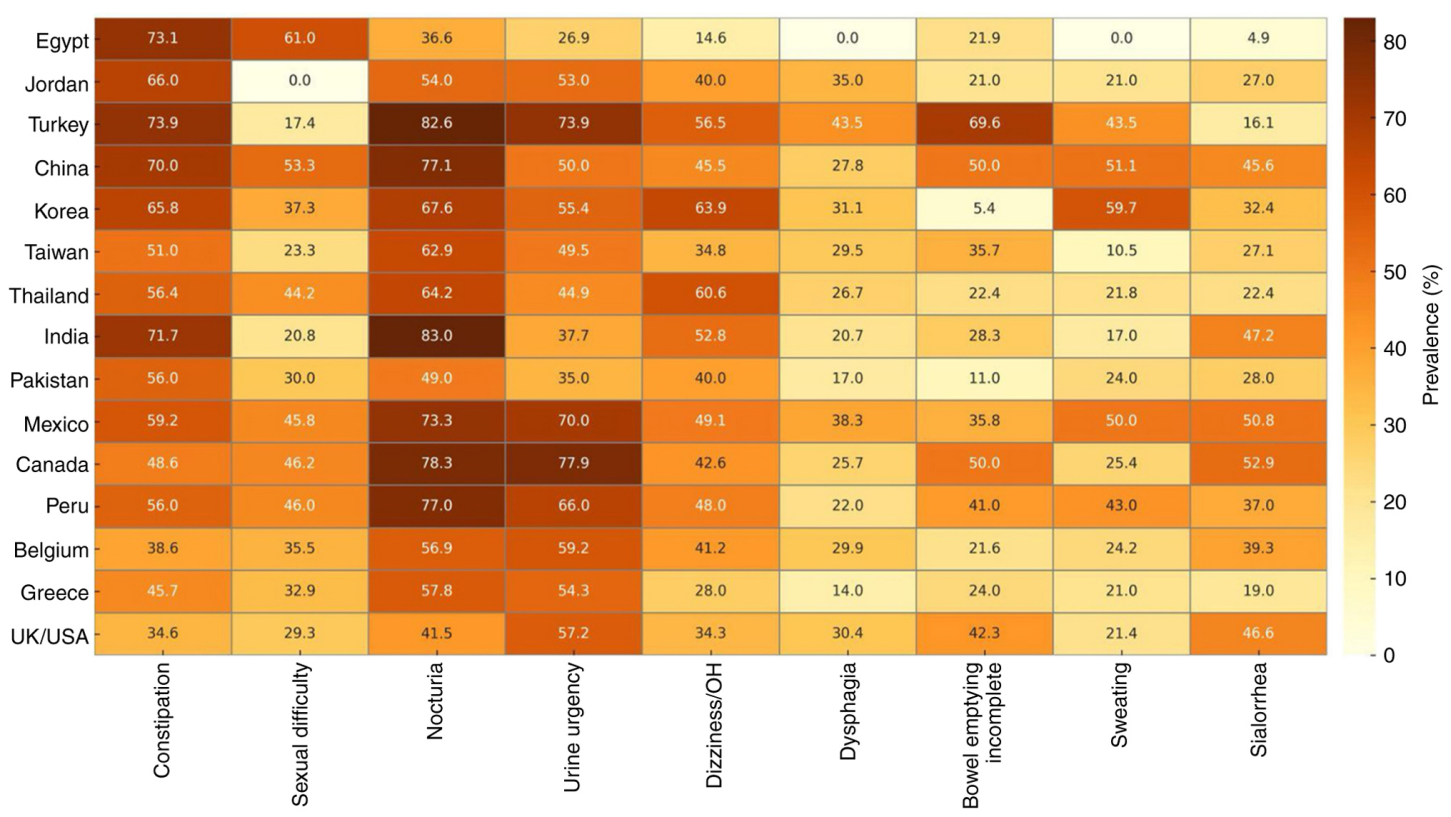
Heatmap illustrating the prevalence of the most common autonomic symptoms across countries. Data derived from studies using the Non-Motor Symptoms Questionnaire (NMSQuest). Values represent symptom prevalence percentage. Consider reading the supplementary material (Table SIII) for further details regarding the percentages and references.

*Geographic and ethnic variations.* There is limited knowledge about geographic and ethnic variations in autonomic dysfunction. However, some studies have tried to bridge the gap (Fig. 3), and (Tables SIII and SIV) (62-76). For example, Sauerbier *et al* (77) discussed the prevalence of non-motor symptoms in Asian patients. They found that autonomic dysfunction symptoms were common among Asian patients. Constipation, memory impairment and nocturia were the most frequently self-reported symptoms (77). Chen *et al* (78) also found differences in non-motor symptoms between Chinese patients with PD and their fellow Western patients. Okubadejo *et al* (79) found that autonomic dysfunction symptoms were more common among patients of African origin when compared to German patients. Sidahmed *et al* (80) suggested that this difference may be due to genetic or environmental factors. These variations can also be due to cultural norms; for example, Zhou *et al* (37) reported lower sexual symptoms and suggested that it may be due to the hesitation of Chinese patients to answer such questions.

Seasonal variation may influence the reporting and severity of autonomic dysfunction in patients with PD. While some studies have suggested that autonomic symptoms are less prevalent in summer compared to winter (81), this seasonal difference appears to be most pronounced in cardiovascular dysautonomia (82). Additionally, winter months are associated with increased mortality rates in PD, primarily due to respiratory infections, such as pneumonia (83); however, some researchers have only found statistical significance for cardiovascular dysautonomia (84). Notably, the impact of seasonal changes on autonomic symptoms may be partially mediated by their influence on sleep-related disturbances, which are common in PD.

*Disease duration, severity and phenotype.* Studies have found that autonomic dysfunction is more common among patients with a longer disease duration (56,85,86), and with more severe motor symptoms (Hoehn and Yahr Scale scores 4 and 5) (47). Previous studies have also demonstrated that the severity of autonomic dysfunction symptoms is associated with the severity of motor symptoms (47,56), and mainly occurs in individuals with non-tremor dominant PD forms (87). Furthermore, right-sided motor presentation has been shown to be related to a higher frequency of autonomic functions (88); however, other research has not revealed significance with laterality at presentation (89). Moreover, patients with normal neuroimaging characterized by non-dopaminergic loss usually have worsening autonomic features (90,91).

Gu *et al* (92) found a predilection for autonomic dysfunction among specific PD subtypes. Patients with PD with mainly gait impairment more frequently had pupillomotor, thermoregulatory and sexual dysfunction. On the other hand, other phenotypes were more commonly associated cardiovascular issues. Urinary and gastrointestinal dysfunctions were not associated with any specific subtype (92). Wang *et al* (93) found similar results with primary gait impairment being correlated with OH and thermoregulatory abnormalities.

Autonomic dysfunction is not only associated with the severity of the disease, but the early development of autonomic dysfunction has been found to increase the risk of reaching the first disease milestone by 14% per year. It has also been shown to be associated with disability, a shorter survival rate and earlier reaching PD endpoints (23,94). Moreover, patients with PD primarily affected by non-motor symptoms with prodromal symptoms have longer disease duration and worse autonomic dysfunction over time (95), particularly Caucasians (32). In addition, some researchers have found that autonomic dysfunction is part of a cluster of patients with cognitive impairment, psychosis and depression (96).

Longardner *et al* (97) reported that only cardiovascular dysautonomia was associated with cognitive impairment. Apparently, patients in the early stages of PD that have abnormal increased sympathetic response more commonly develop cognitive impairment (98). In addition, some researchers have found an association between the overall autonomic dysfunction and cognitive decline (99).

A high frequency of autonomic symptoms is associated with specific gait features in PD involving decreasing swing, cadence and velocity. Nevertheless, subdomain analysis has demonstrated that only urinary symptoms are directly associated with gait impairment (100). In other studies, freezing of gait (101) and postural instability (102,103) were shown to be directly associated with the overall autonomic dysfunction. However, the association with freezing of gait and autonomic symptoms were not observed in other research (104).

Yoon *et al* (105) found another cluster of patients with hyposmia and autonomic dysfunction with overall lower uptake in dopamine transporter imaging and a higher risk of dementia. However, the risk of motor complications was the same (105). Pan *et al* (106) found that patients with dysautonomia may have more rapid brain atrophy, particularly in the temporal region. A similar subgroup termed ‘biotype 1’ characterized by subcortical volume loss was found by Wang *et al* (107). In addition, an inverse proportionality was found between dopamine transporter single-photon emission computed tomography and overall autonomic symptoms (94).

*Non-motor fluctuations in autonomic symptoms.* Autonomic symptoms in PD are not only persistent, but may also exhibit fluctuations that parallel motor ‘on-off’ states. These non-motor fluctuations include episodic changes in BP, sweating, gastrointestinal motility and urinary urgency. For instance, hyperhidrosis is frequently reported during ‘off’ periods and may improve during ‘on’ states, suggesting a dopaminergic modulation of sudomotor pathways (108,109). Similarly, orthostatic hypotension can worsen during ‘off’ periods due to a reduced sympathetic tone, while some patients experience transient postprandial hypotension that varies with medication timing (110). Gastrointestinal symptoms such as bloating, nausea and constipation may also fluctuate, potentially linked to delayed gastric emptying during ‘off’ states (111). Urinary urgency and frequency have been observed to intensify during periods of reduced dopaminergic stimulation, although evidence remains limited. These fluctuations are often underrecognized, yet they significantly affect QoL and may complicate symptom management (112). Recognizing the dynamic nature of autonomic dysfunction in PD is essential for tailoring treatment strategies and improving patient outcomes.

*Effects of medication.* The study by De Pablo-Fernandez *et al* (23) found that the development of autonomic dysfunction was related to a poorer response to levodopa treatment. It was also associated with lower maximum LEDD (23), although there are contradictory results in the literature (113). Dopamine agonists are theoretically known to worsen the severity of autonomic dysfunction symptoms (47,114); however, some studies have revealed an improvement in heart rate (HR) due to vagal tone response with levodopa administration (115,116). On the other hand, in another study, there was no cardiovascular dysregulation with rasagiline (117); furthermore another study demonstrated that entacapone intake reduced the risk of OH in patients with PD (118). Of note, Malek *et al* did not find the same association with other medications apart from dopamine agonists (56). A previous systematic review found no association between OH and dopamine agonist or monoamine oxidase-B inhibitor medications (119).

Aris *et al* (120) demonstrated that unmedicated patients with PD rarely manifested autonomic symptoms, and those taking medications usually developed symptoms later in the disease course, which they hypothesized of being a side-effect. Similar results were also observed by other authors (121). The autonomic side-effects of medications for PD are presented in Table SV.

*Comorbid conditions.* The occurrence of comorbidities, which are a hallmark of this mostly elderly population affected by PD, strongly complicates the condition of autonomic dysfunction (1). The evaluation and treatment of autonomic dysfunction in PD should take these comorbidities into account, as they may independently cause or exacerbate autonomic symptoms and impact their presentation and severity (36).

Guseva and Zhukova (122) reported that hypertension was associated with the progression of PD and the worsening of motor functions. Similar results were reported by Korchounov *et al* (123), with arterial hypertension being an independent risk factor of dysautonomia. Therefore, autonomic dysfunction may be mainly associated with demographic, but not to PD-related factors.

Several comorbidities common to the PD population are established causes of secondary autonomic failure, which are known to contribute to the symptom complex of PD, including conditions, such as diabetes mellitus (124), various cardiovascular diseases (hypertension) and renal insufficiency (58,125). For example, diabetes mellitus may cause autonomic neuropathy, resulting in OH, constipation and gastroparesis that can be difficult to differentiate from those derived from PD (36,58). Cardiovascular diseases pose particular challenges, and previous hypertension may either coexist with diabetes or contribute to SH, a frequent issue for PD-related autonomic dysfunction (38,41). Moreover, drugs for these cardiovascular comorbidities are likely to exacerbate OH episodes in patients with PD (e.g., diuretics and other antihypertensive agents) (26,38).

Autonomic dysregulation can also be worsened by cerebrovascular disease through alteration in the central control pathways, further aggravating the burden of dysautonomia and the intrinsic neurodegeneration (126). Urinary tract pathology, such as benign prostatic hypertrophy in men, may also produce symptoms of frequency and urgency, indistinguishable from the bladder dysfunction commonly encountered in PD (5,49). Furthermore, psychiatric comorbidities, such as depression and anxiety, which are common in PD, can influence the recognition and reporting of autonomic symptoms of the patient, also affecting QoL (127). Hence, comorbid conditions need to be carefully considered when diagnosing autonomic dysfunction in PD and formulating personal management strategies for these non-motor symptoms (35,36).

*Environmental factors.* A number of environmental factors have been studied with PD. For example, cigarette smoking is a known modifiable risk factor associated with a lower incidence of PD (128). Caffeine is also associated with a lower risk of developing PD (129). Dietary vitamin E has been shown to be a protective factor against PD (130). In a recent study by Dorsey and Bloem (131), environmental toxins, including certain pesticides, industrial chemicals and air pollution, were investigated as triggers in the pathophysiology of PD.

*Genetic factors.* Autonomic dysfunction is more common in familial PD cases when compared to idiopathic PD (58,132). For example, synuclein alpha (SNCA) multiplication is an autosomal dominant trait in familial PD, and it is associated with cardiac sympathetic denervation (133); cardiac sympathetic denervation is also common among symptomatic and asymptomatic carriers of the E46K mutation of the SNCA gene (114). In this context, PARK 20 (SYNJ1 gene) is frequently observed with gastrointestinal and urinary symptoms (134).

It has been found that autonomic dysfunction in general and particularly cardiac sympathetic denervation, are less pronounced in patients with familial PD with parkin RING-between-RING E3 ubiquitin protein ligase (PARK2) (135) and ATPase cation transporting 13A2 (PARK9) (134). Song *et al* (136) reported no difference in autonomic symptoms frequency between PARK2 individuals and idiopathic PD.

Patients with PD who are carriers of leucine rich repeat kinase 2 (LRRK2) have been found to have slightly less gastrointestinal dysfunction and RBD than patients with idiopathic PD (137). Similar results have been observed for cardiac dysautonomia (138). However, other research found no difference in the incidence of autonomic dysfunction between individuals who are carriers of LRRK2 compared to non-LRRK2 individuals (139).

Patients with glucosylceramidase beta-1 (GBA-1) mutation, when compared with idiopathic PD, more frequently complain of cardiovascular (140) and gastrointestinal (141) dysautonomia. More specifically, constipation has been shown to be more statistically significant among the gastrointestinal symptoms reported in GBA-PD (142). In addition, GBA-PD has been shown to be associated with a more pronounced involvement of systolic BP than diastolic BP (143).

## 4. Cardiovascular dysfunctions

Cardiovascular sympathetic denervation is common among patients with PD (144), and mild impairment can be noted early in the course of PD (145). The symptoms associated with this dysfunction are OH, postprandial hypotension, SH and non-dipping BP. Nevertheless, half of the patients report non-specific complaints, such as dizziness, lightheadedness, nausea and transient visual impairment (146). In this context, cardiovascular symptoms were not found to be associated with motor fluctuations (147), although they were associated with olfactory dysfunction (148). More specifically, anosmia was associated with a lower baroreflex, a pronounced decrease in systolic BP and a lower increase in norepinephrine during orthostasis (149). A summary of the prevalence of cardiovascular dysfunctions among patients with PD is presented in Table SVI (41,42,150-153).

*OH.* OH is prevalent in ~20% to 40% of patients with PD (150). In drug-naïve patients with PD, OH was found in 11.1% of cases (154). Hiorth *et al* (155) revealed an increase in the prevalence of OH, affecting 65.4% of individuals in 7 years, with only 29.2% exhibiting clinical symptoms. However, the management was uncommon, occurring in only 0.5% of cases (155). The wide variation in prevalence may be attributed to a number of factors, including different populations, inconsistent diagnostic criteria applied, and the type of medications used by the patients (156). In this context, the height or weight of the individual do not influence the incidence of OH in patients with PD (157). However, Umehara *et al* (158) found an association with underweight and cardiovascular dysfunction. On the other hand, Mochizuki *et al* (159) revealed a positive direct assocsation between weight and the risk of cardiovascular dysautonomia.

OH is associated with the duration and severity of the LBD (150), and it is an independent predictor of overall disability (160). Merola *et al* (161) demonstrated that the incidence of OH increased from 31 to ~46% in 1 year Nevertheless, Josta and Augustis (162) found a similar percentage of OH throughout the course of PD, not demonstrating predilection with disease severity.

OH can be asymptomatic or can present with symptoms varying from lightheadedness to a loss of consciousness (156). Among patients with PD, only 43% experience the typical symptoms, whereas 33% are completely asymptomatic (152). OH is associated with a decreased QoL and an increased risk of falls (OR, 10.70) (163), leading to fractures and prolonged peirods of hospitalization (164). Notably, from all the autonomic symptoms, only OH and urinary dysfunction have been associated with increased risk of falls (165), although this has not been reproduced in other research (166). The gait findings that are worse in patients with PD and OH are speed, stride length, postural transition and postural sway (167).

OH is associated with an increased risk of cognitive impairment in executive functions (168). In addition, mild cognitive impairment is related to OH (169), particularly among females (170). Another fact is the white matter changes in patients with PD and OH, which were already linked with cognitive impairment (171). The white matter microvascular changes are mainly observed in the fronto-subcortical and posterior cortical regions (172). Furthermore, the cognitive impairment and the white matter changes are not only linked to cardiovascular dysfunction, but with the overall dysautonomic symptoms (173,174).

Nakamura *et al* (175) noticed that up to 85% of wheelchair-bound individuals with PD were asymptomatic with OH. It would be valuable to have a direct comparison involving wheelchair bound and non-wheelchair-bound individuals with PD regarding their cardiovascular dysfunction.

*Postprandial hypotension.* Postprandial hypotension is a frequent manifestation, affecting up to 60% of patients with PD (176), and it has been found to be independent of meal type (177). Risk factors for postprandial hypotension include OH, constipation and the female sex (178). Consistently, Yalcin *et al* (179) demonstrated that postprandial hypotension affected 94% of patients with OH (153). Diagnosing postprandial hypotension is essential because it is associated with an increased risk of falling. In addition, although postprandial hypotension is more prevalent in individuals with severe PD, levodopa does not influence the hypotensive features (180).

*Non-dipping BP.* Non-dipping BP is having BP that does not decline at night (181), and it is independent of hypertension or anti-hypertensive therapy (182). Non-dipping is prevalent among patients with PD (183). Sommer *et al* (182) demonstrated that non-dipping occurred in 88% of patients with PD and occurred more frequently among patients with OH than those without. Furthermore, it occurred in 100% of patients with SH (182). Similarly, Kapoor *et al* (151) reported that 83% of patients with PD had non-dipping, while 64% had SH. This is clinically relevant, as patients with PD and nocturnal hypertension have a higher rate of left ventricular hypertrophy (184). Furthermore, the non-dipping degree is associated with the severity of PD psychosis (185).

*SH.* SH occurs in ~34% of patients with PD, often in conjunction with OH (41). Goldstein demonstrated that in patients with PD or MSA, OH was associated with significantly higher mean BP at the supine position (109±3 mmHg) compared to patients without OH (96±3 mmHg) (33). In addition, patients with asymptomatic OH had a higher prevalence of SH than symptomatic patients (42). Identifying SH and OH is essential, as they are associated with a higher risk of end-organ damage, cardiovascular events and the overall risk of mortality (186). A previous study reported the case of a patient with posterior reversible encephalopathy syndrome due to the pronounced BP fluctuation and SH (187).

*Others.* Other common cardiac autonomic dysfunction observed with the tilt test in patients with PD apart from OH are chronotropic incompetence (39%) and postural orthostatic tachycardia syndrome (1%) (188). Sebastian *et al* (189) revealed that 47% of individuals with idiopathic PD had a reverse dipping pattern and 80% variability in BP. Milazzo *et al* (190) found reverse dipping in 69% of patients with symptomatic cardiovascular dysfunction. Furthermore, 41% of patients with PD have decreased power spectral analysis of HR variability, indicating early autonomic dysfunction without symptoms (191). Some specialists recommend the early evaluation of cardiovascular function since PD is associated with sudden cardiac death and sudden unexpected death (192). Notably, there is abnormal cerebral autoregulation in PD independent of cardiac dysautonomia (193), and levodopa have been found to increase the local cerebral blood flow (194).

HR variability is affected by PD, and its prevalence is unknown due to the requirement of cardiac testing for its diagnosis (Table SVII) (121,195-202). Abnormal variability was only noted in off-periods with dyskinesia, suggesting that on-periods were associated with a normal HR variability (203). The HR variation is associated with worsening gait impairment and more episodes of freezing of gait (204). However, other researchers have not found significant results on the association between autonomic dysfunction and freezing of gait (205). Carricarte Naranjo *et al* (206) found that the deceleration capacity of HR in patients with idiopathic PD was impaired, and may be an early marker of autonomic failure. In addition, the abnormal cardiac rhythms observed in patients with PD assessed by deep machine learning were diagnostic in up to 90% of cases (207). In another study, the HR variability was shown to be associated with the parasympathetic tone impairment only in a short-term evaluation (208); in another study, there was no significance for long-term measures or worsening of the autonomic function (209). Of note, cardiac rhythm abnormalities may be related to dysautonomia without sinus node dysfunction (210).

Vallelonga *et al* (211) assessed ambulatory BP monitoring with a prevalence of autonomic dysfunction of 36% and accuracy of 91%. During the COVID-19 pandemic, the remote monitoring of cardiac function was effective in the diagnosis and management of OH (212). Notably, the continuous monitoring of BP and HR, compared to episodic, may be more effective in diagnosing the beginning of autonomic failure in PD (213). Apparently, the most effective evaluation of sympathetic and parasympathetic cardiac function in PD is performed by combining the analysis of frequency and time domains of HR (214). Netten *et al* (215) used a device for evaluation of five responses for diagnosis PD and identified 23% of individuals with cardiovascular autonomic failure.

A marker of the cardiac sympathetic dysfunction in PD is OH; however, in the absence of OH, the most effective indirect predictor of sympathetic failure is the low-frequency diastolic BP (216). Park *et al* (217) also reported increased neurofilament light chain levels in patients with PD and OH compared to non-OH.

During traditional aerobic exercise or aerobic exercise performed with self-selected intensity, individuals with PD have been shown to have the same cardiologic response compared to the controls (218). Of note, in another study, a similar response to the controls was observed in patients with decreased hemoglobin levels (219). The stability of the BP may be related to a positive inotropic response against vasodilation even in individuals with impaired cardiac sympathetic function (220). Miyasato *et al* (221) found contradictory results with a lower increase in systolic BP and HR during exercise in patients with PD; this is associated with overt cardiovascular autonomic failure (222) and the magnitude of hypotension is related to the exercise intensity (223). Furthermore, a reduced sympathetic response has been noted during hypoxia without any influence by dopaminergic therapy (224).

## 5. Gastrointestinal dysfunctions

Patients with PD experience gastrointestinal symptoms in the early and prodromal stages of the disease (225). These manifestations include weight loss, sialorrhea, dysphagia, gastroparesis, small intestinal bacterial overgrowth syndrome, constipation and defecatory dysfunction (4). Urinary and gastrointestinal symptoms are the most frequently reported autonomic symptoms, affecting up to 100% of individuals with PD (226).

Dizziness is associated with cardiovascular, but also with gastrointestinal dysfunction (227), although other researchers have not found statistically significant results for these associations (228). Furthermore, apart from the influence of gastrointestinal dysfunction in the cognitive function involving the visuospatial, letter fluency and memory (229,230); olfactory dysfunction is associated with gastrointestinal and urinary dysfunction (231). The prevalence of gastrointestinal dysfunction in PD is summarized in Table SVIII (44,229,232-239).

*Weight loss.* Weight loss is a frequent symptom, affecting almost half of patients with PD (232). The underlying mechanism is not yet understood. However, it may be related to the disease progression rather than reduced appetite (232). Weight loss is associated with malnutrition, falls, fractures and a poor QoL (240). Furthermore, it is associated with cognitive impairment, dependency and mortality (126,241). In addition, patients with early-stage PD that have weight loss usually develop more severe cardinal motor symptoms, cognitive function and executive function; on the other hand, patients with weight gain may have slower impairment in processing speed, attention and motor symptoms (242).

*Sialorrhea and xerostomia.* The frequency of sialorrhea ranges from 32 to 77% in patients with PD (243), with a mean frequency of almost 50% (244). Patients with PD could experience sialorrhea in the early or late stages of the disease (56,233). Müller *et al* (245) found that the most frequently reported autonomic symptom observed in patients with untreated PD at the time of diagnosis was drooling. van Wamelen *et al* (233) demonstrated that following 3 years of follow-up, the frequency of sialorrhea increased from 37 to 40%; however, its severity was not affected. Nevertheless, other authors have found that sialorrhea is associated with an older age and the frequency of other non-motor symptoms (246). Furthermore, sialorrhea is often associated with dysphagia and could be an indicator of subclinical dysphagia (233,247).

Xerostomia is reported in ~50% of individuals with PD (234), with a higher mean prevalence than drooling. But, it is rarely mentioned as an autonomic symptom (248), and only 12% of the patients complain to their physicians of dry mouth (249). Xerostomia is associated with dysphagia, and unfortunately does not improve with drooling. It can severely affect the QoL (250), and it is a commonly side-effect of medications in patients with PD (251). It is worth mentioning that patients complaining of xerostomia have increased concentrations of acetylcholinesterase activity (252).

*Dysphagia.* Dysphagia is a frequent gastrointestinal symptom in patients with PD, and its incidence surprisingly increases after 15 years of the PD onset (253). Gong *et al* (254) found that dysphagia occurred in 36% of patients, varying from 9 to 77%, and Oceania has the highest prevalence followed by Africa, Asia, Europe and America. Moreover, the prevalence was higher when dysphagia was confirmed using instrumental examination, 44 to 69.1% (254). Dysphagia is associated with an older age, disease duration, body mass index, severity of motor symptoms and depression (255). Dysphagia affects the QoL of patients with PD (256). Moreover, it can result in malnutrition, dehydration and aspiration pneumonia (257). Nevertheless, some researchers have not find a strong association between dysphagia and nutritional status (258).

*Gastroparesis.* Gastroparesis is delayed gastric emptying without obstruction on the upper GI system, along with symptoms, such as nausea, fullness, bloating and distension for a period ≥12 weeks (259). One of the clinical findings of decreased gastrointestinal motility is the reduced bowel sounds with digital auscultation that can be observed in patients with PD (260).

Delayed gastric emptying is highly prevalent among patients with PD, affecting ~70 to 100% of this population. However, the exact prevalence of symptomatic gastroparesis remains unknown due to limited data (261). Soykan *et al* (236) reported that among patients with gastroparesis, 7.5% of them had PD. Trahair *et al* (262) observed an inverse association between the gastric delayed time and the blood glucose in PD. Gastroparesis is less prevalent than other gastrointestinal symptoms. However, it is associated with a significantly reduced QoL (263).

*Small intestinal bacterial overgrowth syndrome (SIBO).* SIBO involves an increase in the count of small intestinal bacteria >100,000 cells per ml (264). The prevalence of SIBO in patients with PD is ~36 to 56% (265). SIBO manifests as bloating, abdominal pain and diarrhea (264). Moreover, SIBO is associated with poor motor functions among patients with PD (266). One of the factors associated with a high percentage of SIBO in PD is the prolonged small intestine passage observed in almost 100% of patients (267).

*Constipation.* Constipation is a common autonomic symptom, affecting ~60% of patients with PD (44). Moreover, it affects ~20 to 25% of individuals in the prodromal stage; thus, constipation is considered one of the Movement Disorders Society (MDS) criteria for prodromal PD (268). Furthermore, it is associated with cognitive impairment and apathy among older patients (269,270). Notably, it is an independent factor of the onset of cognitive impairment (271), and more severe motor and non-motor symptoms (44). Some researchers have revealed the occurrence of diarrhea in 28% of patients with PD, although this symptom is not associated with QoL (272).

*Defecatory dysfunction.* Defecatory dysfunction affects ~60% of patients with PD (238). Patients with PD and defecatory dysfunction have impaired squeezing pressure and an impaired recto-anal gradient during defecation (273). Defecatory dysfunction may be the leading cause of constipation in PD. Ramu *et al* (273 reported that among patients with PD and constipation, 40% of them had defecatory dysfunction*.* Zhou *et al* (274) reported that 94% of patients had at least dyssynergia, and 19% of patients reported anal hypotension, 47% hypocontractility, and 8% of patients had both. Krogh *et al* (275) revealed that levodopa therapy was associated with an improvement in the defecatory response.

## 6. Urinary dysfunctions

Urinary and sexual dysfunctions are prevalent among patients with PD. Lower urinary tract symptoms affect up to 89% of patients with PD (276). At the same time, sexual dysfunction affects up to 88% of patients with PD (277). As opposed to other autonomic dysfunctions, evidently, urinary symptoms are not associated with cardiovascular manifestations (278). The prevalence of urinary and sexual dysfunctions in PD is summarized in Table SIX (50,276,279-282).

*Urinary dysfunction.* Urinary dysfunction symptoms can be classified as voiding or storage symptoms. Voiding symptoms involve incomplete emptying and intermittent voiding (279). Storage symptoms involve frequency, urgency and urge incontinence (283). Both voiding and storage are affected in patients with PD, and this can severely affect QoL, particularly at the early stages of the disease (284). These symptoms are associated with an increased risk and recurrence of urinary tract infections (285). Notably, confounding factors of urinary symptoms should be excluded prior to the diagnosis of associating with PD. Valentino *et al* (286) found that 42.8% of patients with PD complaining of lower urinary tract symptoms had benign prostatic hyperplasia.

Micturition disturbances in PD can be formally classified into distinct etiological categories: secondary urinary symptoms, such as those caused by benign prostatic hyperplasia and adverse effects medications; lower urinary tract dysfunction resulting directly from the neurodegenerative process of PD; the infrequent occurrence of detrusor hypoactivity; and the rare presence of detrusor-sphincter dyssynergia. Of note, Galloway *et al* (287) found abnormal sphincter contractions in patients with PD, which is known as ‘urethrismus’ (288). Detrusor hyperactivity can be also be observed in individuals with PD, although no association with motor impairment has been noted (289).

*Sexual dysfunction.* Sexual dysfunction is a prevalent non-motor symptom in PD. However, it is often underreported (290). Sexual dysfunctions involve reduced sexual urge, hypersexuality and ejaculatory or erectile dysfunction (290). It occurs in the late and early stages of the disease (279). Moreover, Durcan *et al* (225) reported the incidence of 15% of sexual dysfunction in the prodromal stage of PD. Yu *et al* (291) found that more than half of individuals had normal sexual fantasy and this was associated with the duration of PD; thus, complaints of sexual dysfunction in patients with PD with advanced disease are clinically relevant.

Sexual dysfunction is associated with age, anxiety and depression (277). Evidently, depression is the main predictor for loss of libido in patients with PD, and this is not influenced by antidepressant therapy (292). Furthermore, it is related to the age of onset of the disease. Özcan *et al* (279) demonstrated that sexual dysfunction was more frequent among patients with late-onset PD (80%) compared to those with early-onset PD (59%). However, available evidence on the association between sexual dysfunction and sex is inconsistent. Shalash *et al* (280) reported that sexual dysfunction was more prevalent among males. Haktanır and Yılmaz (277) reported that it was more common among females. There was a positive association between sexual dysfunction and other autonomic dysfunction, including gastrointestinal (293); however, this finding was not consistent in other research (294).

*Erectile dysfunction*. Shalash *et al* (280) demonstrated that ~70% of male patients with PD experienced erectile dysfunction, and the severity of sexual dysfunction was associated with age. Erectile dysfunction affects the QoL of patients and is associated with cognitive dysfunction (280). Notably, sexual dysfunction in patients with PD is managed with evidence-based drugs (295).

*Female sexual dysfunction*. Females with sexual dysfunction can experience reduced sexual desire, hypersexuality, vaginismus, loss of lubrication, involuntary urination, or orgasm dysfunction (290,296). In this context, all autonomic spheres evidently worsen with the progression of PD, apart from female sexual dysfunction (297). The most prevalent among these symptoms are orgasm dysfunction (50%) and reduced sexual desire (48%) (281); this can negatively affect QoL (298). Furthermore, only a small number of females seek medical advice for sexual dysfunction. Deraz *et al* (298) revealed that only 2% of females sought medical advice compared to 30% of males.

*Restless genital syndrome*. Restless genital syndrome is defined as excessive and persistent arousal of the genitalia not associated with sexual desire. It is often described as tingling, burning, throbbing, or pain. These unwanted feelings may persist for the entire day. Moreover, they are not relieved by ordinary orgasm (299). Although these genital symptoms are considered rare to occur in PD, they could be severe and disabling (300). Aquino *et al* (301) reported a case of PD and disabling genital discomfort in a female aged 65 years.

## 7. Thermoregulatory dysfunction

The first description of thermoregulatory dysfunction as a ‘special sense of coldness in the affected limbs’ in PD can be found in the textbook of Gowers (302) entitled ‘A Manual of Diseases of the Nervous System’ in published 1983. Gowers emphasized the progressive nature of these symptoms and also noted hyperhidrosis on the side of maximum motor impairment. This preferential affection of the side with maximum motor deficits has been reported in later studies as well (108,197).

Thermoregulatory dysfunction in PD arises from both central and peripheral autonomic impairment. Alpha-synuclein pathology and Lewy body formation in the hypothalamus, brainstem nuclei, and spinal preganglionic neurons disrupt central autonomic regulation (303,304). Peripherally, small fiber neuropathy affecting sweat glands, erector pili muscles, and vascular innervation has been reported, and may be related to the length of levodopa exposure (305,306). Neuropathy in PD has also been shown to be associated with vitamin B12 deficiency and the neurotoxic effects of elevated homocysteine and methylmalonic acid levels (307). Several functional studies have reported abnormalities contributing to thermoregulatory impairment in PD. These include asymmetric sympathetic skin response (SSR) dysfunction favoring the more affected motor side (308,309), reduced frequency of skin sympathetic nerve activity observed across disease stages (310,311), diminished cutaneous vasoconstriction (312) and levodopa-associated reductions in venous tone (313).

In general, patients with PD report a spectrum of sweating disturbances, ranging from excess sweating to reduced sweating (Table SX) (6,197,308,314-319). When queried to compare their sweating patterns to prior to their diagnosis of PD, sweating issues were reported in ~2 in every 3 individuals with PD; however, studies report a broad range of these complaints between 10 to 100% of patients (6,314,320). Hyperhidrosis is more commonly reported than hypohidrosis (6).

Siepmann *et al* (321) revealed defects in the pilomotor function, but not in the sudomotor axon-reflex with PD progression, and this effect was associated with motor symptoms. Asahina *et al* (322) found that the sudomotor response impairment was observed even at the diagnosis of PD prior to commencing any medication. In addition, Xu *et al* (323) found association with LEDD and the development of electrochemical skin conductance tests. Nevertheless, no association has been found between seborrheic dermatitis and autonomic dysfunction in patients with PD (324).

Anbalagan *et al* (325) assessed infrared thermography in PD, and they found that the recovery rate was impaired in PD and was dependent on dopaminergic agents and motor impairment. Purup *et al* (326) reported similar findings, although there was no association of the thermography difference with other autonomic dysfunctions. Noteworthy, Rocchi *et al* (327) assessed patients complaining of sudomotor dysfunction, although the electrochemical skin conductance was normal. Similarly, Roy *et al* (328) did not observe abnormality in the standard cold pressor tests.

*Hyperhidrosis.* Hyperhidrosis is mostly episodic in PD and may be generalized or asymmetric, the latter being more common in the side of greatest motor involvement (308,329). In cases when hyperhidrosis is not related to motor impairment, it tends to involve mainly the trunk and the head (6). Hyperhidrosis is more often reported during ‘off’ periods (330), and it can improve during ‘on’ periods, which is known as non-motor fluctuations (109). The study by Pursiainen *et al* (108) demonstrated a significant increase in sweating at the hand site using an evaporimeter in patients with motor fluctuations but not in those without notable ‘off’ symptoms. Increased sweating has also been found problematic in patients with severe dyskinesias, which is considered to result from excessive physical activity (6). The underlying pathophysiology of this possible connection between sweating and motor fluctuations is likely poorly understood and may involve disordered sympathetic activation, inadequate central dopaminergic stimulation, or alterations in dopamine levels (110,197). Hyperhidrosis is also in part deemed to be compensatory to lower body hypohidrosis (315). In this manner, the hypohidrosis of the hands and feet can lead to thoracic (331) and head (332) increased sweating due to reduced sympathetic function in extremities. Selective norepinephrine reuptake inhibitors may also cause hyperhidrosis (333).

**Figure 4:**
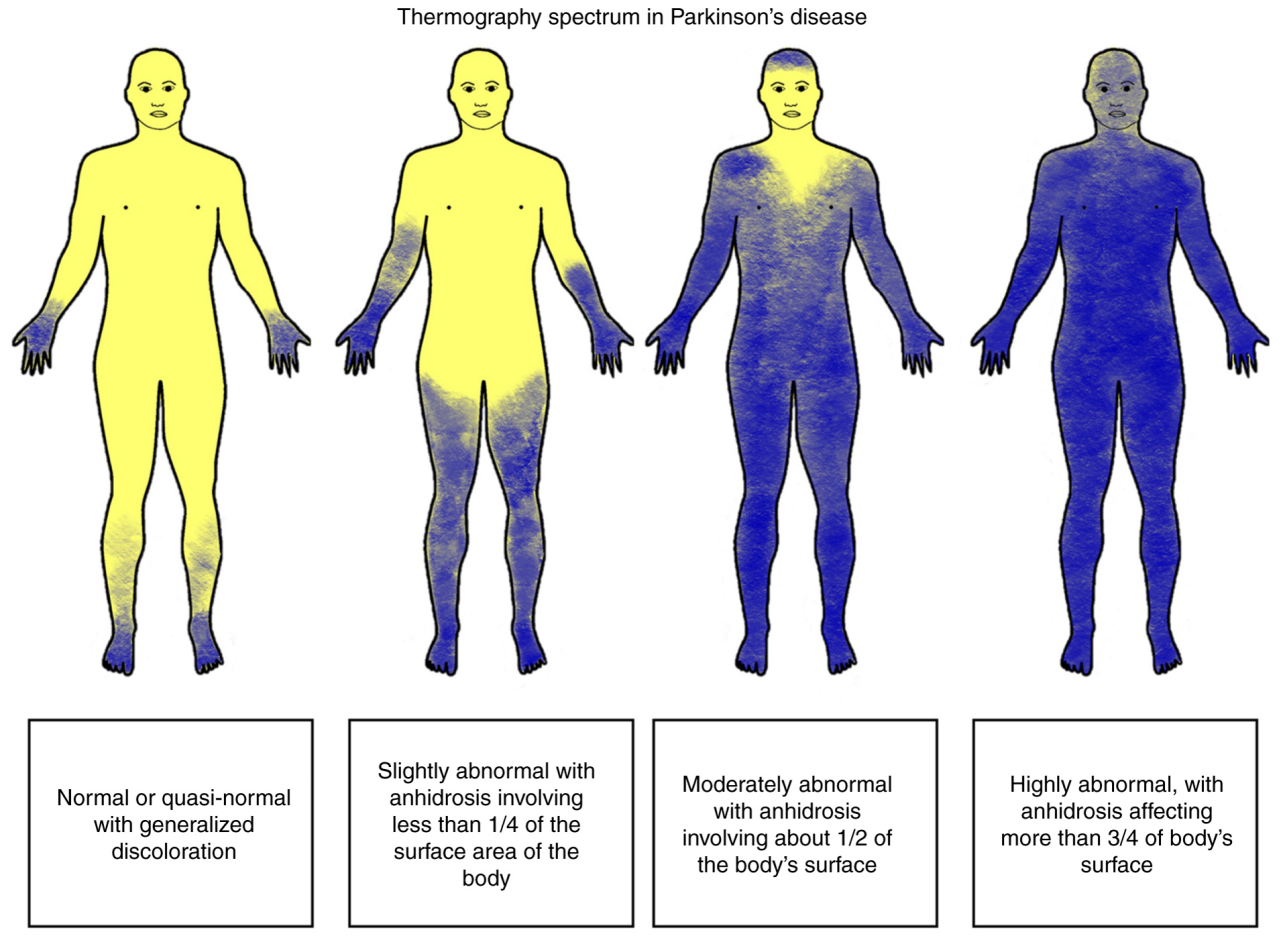
Thermography spectrum in Parkinson's disease. Anhidrosis pattern on thermoregulatory sweat test noticed in Parkinson's disease, by Saito *et al* (319). The pharmacological sweating test is induced by intradermal injection of acetylcholine, and a capacitance hygrometer is used to quantitatively measure the sweating.

*Hypohidrosis.* Hypohidrosis is less commonly reported by patients with PD than hyperhidrosis (6). It is likely due to the fact that the latter is more problematic for patients; however, it is important to note that hyperhidrosis may in part be compensatory to lower-body hypohidrosis (315,334). Decreased sweating is often observed in a length-dependent pattern involving the distal part of the lower extremities (335). The only clinical symptom of hypohidrosis or anhidrosis in PD may be heat intolerance and is reported in approximately two thirds of patients (6). Saito (319) reported that only 30% of the individuals with PD had normal thermoregulatory function, and 9% had severe anhidrosis, affecting >75% of the body. The main part of the body being affected is the lower limbs, and less commonly, the upper extremities and the face (Fig. 4) (319,336). Notably, individuals with tremor-dominant PD are less commonly affected by sudomotor impairment (319). Patients with PD in later stages of the disease, based on Hoehn and Yahr staging, demonstrate a more pronounced reduction in sweat volumes at leg sites compared to patients with early-stage disease, consistent with the established observation that autonomic dysfunction tends to worsen with disease progression (12,337). Hypohidrosis and heat intolerance may also result from the use of anticholinergic medications (336).

*Hypothermia.* Hypothermia is generally described as a body temperature of <35˚C (95˚F). Temperatures as low as 30.9˚C have been reported in the literature in patients with PD, and it mainly occurs during the nighttime (338). Clinically, these patients may present with worsening bradykinesia and rigidity, myoclonus and somnolence or coma (317,339-341). Electroencephalography may show widespread slowing and triphasic discharges (341). Mortality in these hypothermic patients is primarily linked to their comorbid conditions rather than their core temperature (342). Other reported thermoregulatory symptoms in PD are heat or cold intolerance and vasomotor issues such as blue mottling of the skin (336).

*Parkinsonism-hyperpyrexia syndrome.* Parkinsonism-hyperpyrexia syndrome usually results from abrupt discontinuation of anti-Parkinson's medications and can be a fatal, albeit rare, complication. Clinically, it resembles neuroleptic malignant syndrome and is a result of depletion of central dopaminergic transmission (343). Dehydration, hot weather, febrile infection and the concurrent use of neuroleptic medication may all contribute to this syndrome in addition to the withdrawal of dopaminergic medications (344,345).

*Sebum secretion, sympathetic skin response and others.* Another tool for controlling body temperature is sebum production. Kitagawa *et al* (346) found a direct correlation between the sebum production and cardiac sympathetic function.

Sympathetic skin response can be measured by different methods. Ozawa *et al* (347) assessed the electric shock stimulation of the frontal head region, and they found an association between the amplitudes of response and the cardiac sympathetic function. However, other studies have reported that the SSR is not related to cardiac dysautonomia (348) and the duration of disease (349) or motor, behavioral, and cognitive changes in PD (350). It has been shown that sex, medication in use and PD phenotype do not influence SSR (351), even in the presence of other autonomic dysfunctions (352). When specifically analyzing motor symptoms, bradykinesia and rigidity, but not tremor, have been found to be associated with an abnormal skin response (353). Of note, manganism in PD can cause abnormal SSR and RR interval variation (354).

Patients with PD commonly report ‘severe cold in lower limbs’, which mainly occurs during the winter, although this has also been in the summer, and it does not improve with dopaminergic medications (355). The fact that patients with PD have abnormal cold stress responses may support this finding (356). Another phenomenon observed to be impaired in 50% of patients with PD is the skin wrinkling test (357), and is more prevalent in the side most affected by motor symptoms (358).

## 8. Pupillo-motor and tear abnormalities

There are numerous pupillo-motor and tear abnormalities studies on patients with PD. Pupil size is decreased in patients with PD, but also in individuals with prodromal phases such as idiopathic RBD (359). Patients with PD may have a slower peak constriction, and dilation amplitude and velocity, which is worse in the presence of cardiac dysfunction (360). Manohar and Husain demonstrated the resolution of the pupillary reward with dopamine agonists (361). Dietz *et al* (362) found normal pupillary sympathetic responses, although the movement of the eyes was slow. Hori *et al* (363) revealed abnormal parasympathetic response and pupillary supersensitivity to light.

There is an association between spontaneous changes in pupil diameter and motor symptoms in PD (364). In addition, patients with PD are more sensitive to the instillation of parasympathomimetic and sympathomimetic agents (363); however, this finding is not associated with disease duration (365). Furthermore, abnormal tear function has been noted in patients with PD, and it is more commonly observed in patients with severe PD (366,367). In this context, as demonstrated in a previous study, patients with PD have half of the quantity of tears than the controls, which may be related to autonomic and emotional disturbances (368). However, other researchers have not found an association with the pupillary abnormalities and the severity of PD (369). Autonomic dysfunction is related to corneal nerve loss in PD by unclear mechanisms, mainly in individuals reporting gastrointestinal and urinary symptoms (370).

## 9. Sleep autonomic dysregulation

Sleep disorders affect 40 to 90% of patients with PD (371). Abnormalities in the autonomic parts of the sleep is observed in 23% of the individuals (372), and they are mainly characterized by abnormal breathing patterns (373). The most common abnormal respiratory sleep pattern is tachypnea with awakening, and central and obstructive apnea in patients with other autonomic dysfunctions (374). In addition, the fact that there is impairment of motor function and arousal mechanisms can lead to further abnormalities in sleep (375).

In general, the severity of autonomic dysfunction is correlated with prevalence of sleep disorders (85,376-378). Wüllner *et al* (379) found that sleep abnormalities were more common in females, and there was an association of their severity and the disease duration. Another study also revealed an inverse association with HR variability during sleep and motor symptoms severity (380), during REM and non-REM sleep (381). Notably, patients with idiopathic RBD that later develop PD have more commonly autonomic dysfunction, particularly nocturia and sleep fragmentation (382) or gastrointestinal motility impairment (383).

OH and constipation are the main dysfunctions associated with sleep behavioral disorders, and the atonic time during sleep is directly associated with OH (384). Fujita *et al* (385) reported that urinary and cardiovascular domains were associated with sleep disorders in PD. Matsubara *et al* (386) reported an association between sleep issues and only nocturia in a stepwise regression, which indicates that there is overlap among autonomic symptoms as a factor associated with sleep.

Excessive daytime sleepiness (EDS) was associated with autonomic dysfunction, in a bidirectional model, suggesting that the treatment of EDS may lead to an improvement of autonomic functions (387). However, there is no association with all the autonomic domains; for example, OH is not associated with EDS (388).

Cho *et al* (389) studied some autonomic features of the sleep architecture, and they found that patients with PD and idiopathic RBD had abnormal activation, and this finding was not associated with sleep spindles. Thermoregulatory response has also been normally observed during sleep in patients with PD (390).

## 10. Impact on quality of life

The definition of QoL, according to the World Health Organization (WHO), is as follows ‘an individual's perception of their position in life in the context of the culture and value systems in which they live and in relation to their goals, expectations, standards, and concerns’ (391). QoL encompasses physical, psychological, autonomy, cognitive, social relations and environmental factors (391).

Numerous aspects of QoL (activity of daily living, emotion, cognitive functions, communication and social support) are affected by autonomic dysfunctions (392). In addition, autonomic symptoms are among the nonmotor symptoms with highest influence in QoL (393). In this context, there is an association between the emotional disturbances and the autonomic dysfunction, which can significantly impact the QoL (394). Of note, dysautonomia is associated with the worsening of depression (395) and fatigue (396) in individuals with PD. Depression is directly associated with autonomic symptoms independently of the motor symptoms in patients with PD (397), mainly constipation and sensation of residual urine (398). In addition, the bidirectional association is also observed with OH and depression (399). Some studies have evaluated this association and found that activities of daily living is the confounding factor between these variables (400). On the other hand, fatigue is associated with gastrointestinal, urinary and cardiovascular dysfunction (401); notably, the strongest factor related to fatigue is the presence of OH (202). Nevertheless, some reports describe no influence in fatigue by autonomic symptoms (402).

Autonomic dysfunction symptoms significantly impair the QoL scores of patients with PD, even more than motor symptoms (403,404). Gastrointestinal symptoms are among the autonomic domains most likely associated with this finding (405). Magerkurth *et al* (406) reported that only bladder dysfunction significant impaired QoL. Notably, in another study, logistic regression analysis did not reveal an association between autonomic symptoms and QoL (407). The PRIAMO study revealed that urinary and gastrointestinal symptoms are the most common autonomic and among the five most frequently reported nonmotor symptoms that negatively affect QoL (408).

*Functional impairment.* The majority of patients with PD are cared for by friends or family, who also suffer from significant psychological burden, and issues that affect their socioeconomic, mental, and physical well-being. The QoL of caregivers tends to be reportedly decrease due to these factors, which affects patient care (409). Furthermore, the increased caregiving burden and increased illness severity over time are key factors for the institutionalization of patients with PD (410), particularly in individuals that develop cardiovascular dysautonomia (411). Grün *et al* (412) found that autonomic dysfunction was the strongest factor contributing to the burden of caregivers. Of note, institutionalized individuals with PD suffer from severe impairments in motor and cognitive functions, and activities of daily living (413).

*Psychosocial impact.* Patients with PD are highly susceptible to developing psychiatric comorbidities. A previous study found that 36% of patients had depression, 33% had anxiety, 40% had fatigue and 47% had sleep disturbances (414). Social isolation is more common among those who suffer from depression symptoms (415). Notably, 21% of patients with PD are also affected by impulsive control behaviors. In this context, autonomic dysfunction was directly associated with this neuropsychiatric symptom (416).

Autonomic dysfunction also interferes significantly with the social life of patients with PD. For example, a previous study demonstrated that patients with urinary incontinence reported significantly higher levels of depression and stress and lower levels of self-esteem compared to healthy controls (417). Urinary incontinence was significantly associated with impairment in activities of daily living, disability and less social network integration (418). Apart from urinary symptoms, other dysautonomic manifestations can affect the social life of patients with PD. For example, the drooling of saliva becomes more prominent with hyperkyphosis, and the tendency for the mouth to remain open often causes social anxiety in patients with PD (419).

## 11. Diagnostic challenges and future directions

Despite the high prevalence and significant impact of autonomic symptoms in PD, epidemiological studies investigating these symptoms remain markedly limited. This gap may be attributed to multiple interrelated factors. Clinician awareness of autonomic dysfunction is often limited, and even when recognized, assessment is hindered by a lack of standardized, validated diagnostic tools and clear definitions. Autonomic symptoms often overlap with those observed in other parkinsonian and non-parkinsonian disorders, such as MSA, LBD and diabetic autonomic neuropathy, which often delays proper diagnosis. As researchers over the past decades have tended to focus on the motor manifestations of PD, non-motor symptoms have been historically neglected, including those related to autonomic dysfunction. In addition, a number of patients underreport these symptoms, either as they perceive them as unrelated to PD, are not significantly bothered by them (e.g., hypohidrosis), or may feel anxious or hesitant discussing issues such as sexual dysfunction.

The scarcity of autonomic specialists and limited exposure to dysautonomia during neurology training further exacerbate the underrecognition of these clinical symptoms. Even when recommended tools are available, such as those endorsed by the International Movement Disorder Society, they are often underutilized. Numerous of these scales are time-consuming, require multiple instruments to assess different symptoms, and are thus difficult to integrate into busy clinical workflows, contributing to under-diagnosis and misdiagnosis. Numerous providers may also not feel confident to assess these symptoms as they may not be familiar with these presentations or may not know how to recognize them.

A 2009 task force from the Movement Disorder Society systematically reviewed rating scales for sialorrhea, dysphagia and constipation in PD (420). Their findings reinforce a number of the challenges outlined above. Of the disease-specific scales, none met the full criteria for ‘Recommended’ status, with the majority falling into the ‘Suggested’ category due to limited validation. For example, the Drooling Severity and Frequency Scale (DSFS), the Swallowing Disturbance Questionnaire (SDQ) and the Sialorrhea Clinical Scale for Parkinson's Disease (SCS-PD) are among the most commonly used, but each has either limited psychometric testing or insufficient use beyond the original validation study. No scale for constipation met even the ‘Suggested’ designation due to a lack of validation in PD populations, despite the widespread clinical use of tools such as the Rome III Constipation Module. Among global tools, the Scales for Outcomes in Parkinson's Disease-Autonomic (SCOPA-AUT) and the Non-Motor Symptoms Questionnaire for Parkinson's Disease (NMSQuest) were the only instruments to receive a ‘Recommended’ designation, due to their broader use and more robust validation. The Non-Motor Symptoms Scale (NMSS) was rated as ‘Suggested’, as it exhibits promising clinimetric properties, but has not yet been widely adopted outside of the original studies. These broader tools, however, provide limited granularity in symptom tracking and treatment response, and were not designed specifically for gastrointestinal autonomic dysfunction. The task force concluded that while new scales may not be immediately necessary, existing instruments should be more thoroughly validated, particularly for their use in PD populations, and applied more consistently in both clinical and research settings (420). A summary of the scales developed for the evaluation of autonomic symptoms in patients with PD is provided in Table SXI (72,86,197,421-427).

The underrepresentation of autonomic symptoms in PD research and care warrants a multidisciplinary approach and increased awareness through improved clinician education, the broader use of validated scales and patient-centered evaluation frameworks. For example, cardiovascular abnormalities have a significant impact in clinical practice, and when associated with side-effects related to medication in patients already susceptible to developing abnormalities, this may lead to poor adherence to therapy and worse outcomes (428). In addition, patients usually do not complain of autonomic symptoms in the early stages of the disease, and the diagnosis can only be performed with cardiovascular reflex tests (429). It is worth mentioning that a number of these individuals will not be referred to the neurologist; instead, they will likely have their first visit with the family medicine doctor, cardiologists and urologists (430,431). Therefore, the development of links between the neurologists and these specialties needs to be reinforced, particularly in cases concerning neurodegenerative conditions. Moreover, some autonomic specialists recommend that all patients with PD should undergo a bedside evaluation of BP and HR (432,433).

Biomarkers have been studied in autonomic dysfunction, and positive results were found with thermoregulatory, gastrointestinal and urinary dysfunctions (434). However, it is recommended to perform first cardiovascular autonomic neuropathy (CAN) risk score before more sophisticated tests (435), particularly in patients with PD aged >65 years (79).

Most autonomic dysfunctions in patients with PD are treated with the same algorithm as other conditions. It is possible that these individuals may benefit from specific approaches, as indicated by Panicker *et al* (436). Some evidence for this hypothesis is the fact that patients with PD undergoing deep brain stimulation (DBS) were occasionally found to exhibit an improvement of thermoregulatory (437), urinary (438), gastrointestinal (439) and cardiovascular functions (440-443). Most common location of stimulation was the subthalamic nucleus (444,445). However, the benefits in the autonomic function were not consistent throughout the literature (446,447). Some studies have reported a temporary effect of DBS in autonomic symptoms (448), while others have revealed that the benefit may be related with the amount of daily activity leading to improvement of cardiac autonomic symptoms (449). Cani *et al* (450) found that OH in patients with DBS taking and not taking levodopa had different responses.

It is a common conception that autonomic dysfunction is mainly observed in patients with atypical parkinsonism. Grażyńska *et al* (451) reported that there was no difference between autonomic and neuropsychiatric manifestations in patients with atypical parkinsonism when compared to those with idiopathic PD.

Another critical aspect that warrants investigation is the association between autonomic symptoms and cognitive function. It is well-known that patients with PD can develop impairment of cognition over time, although it is interesting to note that cardiovascular dysautonomia was already associated with cognitive impairment. There is recent evidence to indicate that not only cardiovascular dysautonomia, but overall dysautonomia is associated with cognitive decline in individuals with PD, particularly in de novo cases (452). Apparently, only PD dementia is related to dysautonomia, when compared to Alzheimer's disease, vascular dementia and dementia with Lewy bodies (453). Of note, there is a cluster of autonomic symptoms (gastrointestinal, sexual dysfunction, thermoregulatory and urinary) that worsen together with the progression of PD (297). Nevertheless, some authors have reported no association between cognitive function and autonomic function, and they claimed that these two spheres may progress independently from each other (454).

A critical limitation for the development of new therapies for autonomic dysfunction in PD is unreliable animal models. The majority of the current models are based on indirect findings and do not reflect the pathophysiology observed in patients with PD. In this way, animal studies may lead to results with unknown clinical significance and should be cautiously analyzed (455). Further research with the development of synuclein disease and specific observations of time required to clinical manifestations is warranted.

## 12. Conclusions

Autonomic dysfunction is a common, yet frequently underrecognized component of PD, with symptoms that often precede motor onset and contribute significantly to disease burden. The present review underscores the complex and heterogeneous nature of dysautonomia in PD, shaped by factors, such as age, disease duration and phenotype. Early autonomic involvement is linked to faster clinical decline and increased risk of cognitive impairment, yet these symptoms are often underreported or misattributed.

These findings highlight the need for proactive screening, tailored diagnostic tools and PD-specific treatment strategies. Addressing autonomic dysfunction holistically, alongside motor symptoms, may improve quality of life and potentially alter disease progression. Future research is required to prioritize the development of targeted therapies, explore the interplay between autonomic and cognitive domains, and refine animal models to better reflect the clinical spectrum of PD.

## Supplementary Material

Summary of autonomic dysfunction features across neurodegenerative disorders [adapted from the study by Niimi *et al* (16)]^a^.

Data of the radar chart.

Prevalence of NMSQuest in different studies from different countries.

Prevalence NMSQuest.

Autonomic side-effects of medications for Parkinson’s disease^a^.

Prevalence of cardiovascular dysfunction In Parkinson’s disease.

Detailed overview of autonomic function test findings in Parkinson’s disease across selected studies.

Prevalence of gastrointestinal dysfunction in Parkinson’s disease.

Prevalence of urinary and sexual dysfunctions in Parkinson’s disease.

Prevalence of thermoregulatory dysfunction in Parkinson’s disease.

Clinical scales specifically designed for Parkinson’s disease.

## Data Availability

Not applicable.
