# Supplementary material for: Epidemiology of autonomic dysfunction in Parkinson's disease (Review)
Source: Med Int (Lond). 2025 Sep 1;5(6):68. doi: 10.3892/mi.2025.267 (PMC12464526; doi:10.3892/mi.2025.267)
Supplement: Summary of autonomic dysfunction features across neurodegenerative disorders [adapted from the study by Niimi et al (16)]a. [file Supplementary_Data.pdf]

Table SI. Summary of autonomic dysfunction features across neurodegenerative disorders [adapted from the study by Niimi *et al* (16)][a].

Autonomic feature	Normal	Pure autonomic failure (PAF)	Autonomic failure in MSA (AF-MSA)	Autonomic failure in PD (AF-PD)
BP response to head-up tilt	Stable	↓↓	↓↓	↓/↓↓
HR response to head-up tilt	↑	↔	↔	↔/↑
Baseline plasma norepinephrine	Normal	↓↓	Normal/↓	Normal/↓
Norepinephrine change with tilt	↑	↔	↓/↔	↔
AVP response to tilt	↑	↓	↓	↔
NE receptor sensitivity	Normal	↑↑	↑/Normal	↑/Normal
Bladder dysfunction	None	+/++	++	+
Obstructive urinary symptoms	None	+	++	+
Irritative urinary symptoms	None	+	++	+

[a]Please see the main manuscript and reference list for the reference citations. BP, blood pressure; HR, heart rate; NE, norepinephrine; AVP, arginine vasopressin; MSA, multiple system atrophy; PD, Parkinson’s disease; ↓, decreased; ↑, increased; ↔, no change.

Table SII. Data of the radar chart.

Movement disorder	Incidence (/100,000)	Age of onset (lower quartile)	Disease duration (upper quartile)	Autonomic dysfunction (mean)	Male (%)
PD	17	65	22	80	60
MSA	7.2	50	10	100	59.6
CBD	0.4	45	8	15	55.0
PSP	2.6	65	12	30	48.5
LBD	3.5	50	5	50	54

CBD, corticobasal degeneration; LBD, Lewy body dementia; PD, Parkinson's disease; PSP, progressive supranuclear palsy.

Table SIII. Prevalence of NMSQuest in different studies from different countries.

Country	Prevalence	First author, year of publication (Refs.)[a]
Africa		
Egypt	Constipation (73.1%), sexual difficulty (61.0%), nocturia (36.6%), urine urgency (26.9%), and urine frequency (21.9%)	Ragab, 2019 (62)
West Asia		
Jordan	Constipation (66%), nocturia (54%), urinary urgency (53%), dizziness/OH (40%), dysphagia (35%)	Dahbour, 2022 (63)
Turkey	Nocturia (82.6%), constipation (73.9%), urgency (73.9%), bowel emptying incomplete (69.6%), and OH/dizziness (56.5%)	Sengul, 2015 (64)
East Asia		
China	Nocturia (77.1%), constipation (70.0%), sexual difficulty (53.3%), sweating (51.1%), and bowel emptying incomplete (50%)	Yu, 2010 (65)
Korea	Nocturia (67.6%), constipation (65.8%), OH/dizziness (63.9%), sweating (59.7%), and urgency (55.4%)	Cheon, 2008 (66)
Taiwan	Nocturia (62.9%), constipation (51.0%) urgency (49.5%), bowel emptying incomplete (35.7%), and OH/dizziness (34.8%)	Liu, 2015 (67)
Thailand	Nocturia (64.2%), OH/dizziness (60.6%), constipation (56.4%), urgency (44.9%), and sexual difficulty (44.2%)	Vongvaivanich, 2014 (68)
South Asia		
India	Bowel emptying incomplete (83.10%), constipation (78.5%), urine urgency (76.9%), OH/dizziness 72.3%, and nocturia (67.7%)	Rukmini Mridula, 2015 (69)
Pakistan	Constipation (56%), nocturia (49%), dizziness/OH (40%), urinary urgency (35%), sexual dysfunction (30%)	Mukhtar, 2018 (70)
Central America		
Mexico	Nocturia (73.33%), urinary urgency (70%), constipation (59.16%), sialorrhea (50.83%), and hyperhidrosis (50%)	Sánchez-Martínez, 2019 (71)

North America		
Canada	Nocturia (78.3%), urgency (77.9%), sialorrhea (52.9%), bowel emptying incomplete (50.0%), and constipation (48.6%)	Romenets, 2012 (72)
South America		
Peru	Nocturia (77%), urinary urgency (66%), constipation (56%), bowel emptying incomplete (41%), dizziness/OH (48%)	Cosentino, 2013 (73)
Europe		
Belgium	Urgency (59.2%), nocturia (56.9%), dizziness/OH (41.2%), constipation (38.6%), and sexual difficulty (35.5%)	Crosiers, 2012 (74)
Greece	Urgency (54.3%), nocturia (51.8%), constipation (45.7%), sexual difficulty (32.9%), dizziness/OH (28.0%)	Bostantjopoulou, 2013 (75)
80% UK and 20% USA	Urgency (57.2%), sialorrhea (46.6%), urinary frequency (42.3%), nocturia (41.5%), and constipation (34.6%)	Rodriguez-Blazquez, 2021 (76)

The table demonstrates the prevalence among different studies of autonomic symptoms obtained from the Non-Motor Symptoms Questionnaire (NMSQuest). The literature was searched with the terms ‘Non-Motor Symptoms Questionnaire’ (and) Country. This variable varied according to the country. [a]Please see the main manuscript and reference list for the reference citations.

Table SIV. Prevalence NMSQuest.

Country	Constipation	Sexual difficulty	Nocturia	Urine urgency	Dizziness/OH	Dysphagia	Bowel emptying incomplete	Sweating	Sialorrhea
Egypt	73.1	61.0	36.6	26.9	14.6	0	21.9	0	4.9
Jordan	66.0	0	54.0	53.0	40.0	35.0	21.0	21.0	27.0
Turkey	73.9	17.4	82.6	73.9	56.5	43.5	69.6	43.5	16.1
China	70.0	53.3	77.1	50.0	45.5	27.8	50.0	51.1	45.6
Korea	65.8	37.3	67.6	55.4	63.9	31.1	5.4	59.7	32.4
Taiwan	51.0	23.3	62.9	49.5	34.8	29.5	35.7	10.5	27.1
Thailand	56.4	44.2	64.2	44.9	60.6	26.7	22.4	21.8	22.4
India	71.7	20.8	83.0	37.7	52.8	20.7	28.3	17.0	47.2
Pakistan	56.0	30.0	49.0	35.0	40.0	17.0	11.0	24.0	28.0
Mexico	59.2	45.8	73.3	70.0	49.1	38.3	35.8	50.0	50.8
Canada	48.6	46.2	78.3	77.9	42.6	25.7	50.0	25.4	52.9
Peru	56.0	46.0	77.0	66.0	48.0	22.0	41.0	43.0	37.0
Belgium	38.6	35.5	56.9	59.2	41.2	29.9	21.6	24.2	39.3
Greece	45.7	32.9	57.8	54.3	28.0	14.0	24.0	21.0	19.0
UK/USA	34.6	29.3	41.5	57.2	34.3	30.4	42.3	21.4	46.6

NMSQuest, Non-Motor Symptoms Questionnaire; OH, orthostatic hypotension.

Table SV. Autonomic side-effects of medications for Parkinson's disease[a].

Medication	Orthostatic hypotension[b]	Constipation[c]	Urinary retention[d]
Levodopa	None ↑ (dose-dependent)	+	None (but disease-related)
Pramipexole	↑ (common)	+	Rare
Ropinirole	↑ (common)	+	Rare
Trihexyphenidyl	+	++	++
Benztropine	+	++	++
Amantadine	+	+ (or diarrhea in some)	+
Entacapone	None ↑ (rare)	+ (or diarrhea)	None
Rasagiline	None ↑ (rare)	+	Rare
Selegiline	None ↑ (rare)	+	Rare
Rivastigmine	Can improve	Diarrhea	None (may help voiding)

[a]Data were obtained from https://reference.medscape.com/drugs (accessed on May 17, 2025). [b]Orthostatic hypotension: Dopamine agonists (e.g., pramipexole, ropinirole) and amantadine commonly cause this. monoamine oxidase-B inhibitors and levodopa can cause this as well, particularly in advanced disease or in combination therapy. [c]Constipation: Common in Parkinson's disease itself; however, anticholinergics (trihexyphenidyl, benztropine) and dopaminergic meds can worsen it. dUrinary retention: The majority of medications do not directly cause this, but anticholinergics (trihexyphenidyl, benztropine) are well-known culprits.

Table SVI. Prevalence of cardiovascular dysfunction In Parkinson's disease.

First author, year of publication	Sample size	Age (mean)	Sex (male)	Prevalence	Comments	(Refs.)[a]
Arbogast, 2009	205	71	55.8%	Typical OH symptoms: 43%; atypical OH symptoms: 24%; asymptomatic OH: 33%	A third of patients with severe OH are entirely asymptomatic.	(152)
Velseboer, 2011	1620	NA	NA	27.7%	Association between OH and the risk of PD.	(150)
Palma, 2015	210	66.3	57.6%	OH (according to 20/10 mmHg): 50%; OH (according to 30/15 mmHg): 30%. Symptomatic OH (according to 20/10 mmHg): 16%; symptomatic OH (according to 30/15 mmHg): 13%	The prevalence of OH in PD is high. However, not all patients have symptoms of organ hypoperfusion.	(42)
Fanciulli, 2016	197	70.6	62%	SH: 34%	SH is associated with cardiovascular comorbidities in PD.	(41)
Yalcin, 2016	84	73	45.2%	OH: 40.5%; SH: 23.8%; postprandial hypotension: 47.6%	OH should be evaluated using BP measurements and cardiac autonomic tests with electrocardiography.	(153)
Kapoor, 2023	60	62.3	58%	Non-dipping: 83%; SH: 53%	SH is common among Indian patients with PD.	(151)

[a]Please see the main manuscript and reference list for the reference citations. BP, blood pressure; NA, not available/ not reported; OH, orthostatic hypotension; PD, Parkinson's disease; SH, supine hypertension.

Table SVII. Detailed overview of autonomic function test findings in Parkinson's disease across selected studies.

First author, year of publication	R-R interval at rest	R-R interval during hyperventilation	HR response to active standing	HR response during valsalva maneuver	Orthostatic BP regulation	(Refs.)[a]
Sachs, 1985	NA	Markedly reduced	Within normal limits	Preserved	Preserved	(195)
Goetz, 1986	NA	NA	NA	Blunted HR response	Maintained	(197)
Camerlingo, 1987	Normal	Significantly reduced	Normal	Normal	Preserved	(196)
Ludin, 1987	Within normal range	Reduced coefficient of variation of R-R ratio	Normal	Normal	Normal	(198)
Turkka, 1987	Preserved	Diminished	Attenuated	Significantly reduced	Intact	(199)
Piha, 1988	Variable (normal to reduced)	NA	NA	Abnormal	Variable response	(200)
Meco, 1991	Reduced	NA	Diminished	Diminished	Impaired	(201)
van Dijk, 1993	Normal	Normal	Impaired	Normal	Abnormal BP regulation	(121)
Ahn, 2020	Reduced	NA	NA	Reduced	OH prevalent in fatigue group	(202)

[a]Please see the main manuscript and reference list for the reference citations. BP, blood pressure; HR, heart rate; NA, not available/not reported.

Table SVIII. Prevalence of gastrointestinal dysfunction in Parkinson's disease.

First author, year of publication	Sample size	Age (mean)	Sex (male)	Prevalence	Comments	(Refs.)[a]
Soykan, 1998	146	45.50	18%	Gastroparesis: 7.5%	A good response to pharmacological treatments could be expected among patients with gastroparesis related to viral or Parkinson's disease.	(236)
Cersosimo, 2013	129	64.69	52.7%	Gastrointestinal symptoms: 100%; Constipation: 53.6%; defecatory dysfunction: 61.2%	The most prevalent gastrointestinal symptoms were sialorrhea, dysphagia, constipation, and defecatory dysfunction.	(238)
Sung, 2014	54	67.10	40.7%	Small bowel symptoms: 88.9%; sialorrhea: 24.1%; constipation: 46.3%; excessive strain during defecation: 33.3%	Gastrointestinal dysfunction is highly prevalent in early PD.	(237)
Barbe, 2017	100	71.00	72%	Dysphagia: 47%; sialorrhea:77%	Patients with PD suffered from sialorrhea and dysphagia that impaired their oral hygiene-related QoL.	(234)
Mohamed, 2018	54	62.30	70.4%	Sialorrhea:68.5%; dysphagia: 40.7%	Dysphagia is a frequent symptom in early PD and is significantly correlated with PD phenotype.	(235)
Yu, 2018	306	62.79	54.6%	Constipation: 61.4%	Depression, anxiety, and autonomic dysfunction were associated with a higher risk of constipation among PD patients.	(44)
Cersosimo, 2018	144	65.10	53.4%	Weight loss: 48.6%	Weight loss occurred in nearly half of PD patients.	(232)
Qin, 2019	108	67.97	54.6%	Dry mouth 59.2%, constipation 49.0%, and dysgeusia 37.04%	Being male is a risk factor for gastrointestinal symptoms.	(239)

Jones, 2020	423	61.20	65.5%	Hard stool: 52.4% and constipation 32.5%	Association between gastrointestinal symptoms and cognition	(229)
van Wamelen, 2020	728	65.72	63.5%	Sialorrhea: 37.2%	Sialorrhea was associated with reduced QoL.	(233)

[a]Please see the main manuscript and reference list for the reference citations. PD, Parkinson's disease; QoL, quality of life.

Table IX. Prevalence of urinary and sexual dysfunctions in Parkinson's disease.

First author, year of publication	Sample size	Age (mean)	Sex (male)	Prevalence	Comments	(Refs.)[a]
Zhang, 2015	91	68.32	67%	Urinary dysfunction: 55%	Nocturia is a common urinary symptom in participants with PD.	(50)
Özcan, 2016	88	67.7	61.4%	Sexual dissatisfaction:75%	Sexual dysfunction occurs more frequently and more severely in late-onset PD.	(279)
Lee, 2018	163	68.9	47.4%	Patients with post-void residual urine: 10.42%	Voiding dysfunction is associated with end-organ damage and other non-motor dysfunctions.	(282)
Xu, 2019	100	65.97	55%	Lower urinary tract dysfunction: 89%; frequency: 86%; urgency: 50%; urge incontinence:34%	Patients with PD and overactive bladder had an older age.	(276)
Shalash, 2020	40	56.65	100%	Erectile dysfunction: 70%	Sexual functions are worse among male patients.	(280)
Kinateder, 2022	861	57.3	50.3%	Sexual dysfunction: 52.1%	Sexual dysfunction is a common but underreported problem in patients with PD.	(281)

[a]Please see the main manuscript and reference list for the reference citations. PD, Parkinson's disease.

Table SX. Prevalence of thermoregulatory dysfunction in Parkinson's disease.

First author, year of publication	Sample size	Age (mean)	Sex (male)	Prevalence	Comments	(Refs.)[a]
Appenzeller,1971	25	64.8	44%	Sweating dysfunction: 68%	Majority of patients (68%) demonstrated anhidrosis over trunk and limbs with compensatory hyperhidrosis on face	(315)
Goetz, 1986	32	61	50%	Skin temperature and sweating abnormalities: 100%	Transient thermoregulatory dysfunction was found in all tested patients before medication and normalized afterward suggesting a possible dopaminergic role in thermoregulation	(197)
De Marinis, 1993	22	63.6	59%	Sweating dysfunction: 100%	While there were differences in thermoregulatory dysfunction between the two patient groups, idiopathic PD and parkinsonism with other features, thermoregulatory testing alone cannot definitively differentiate between them in the early stages.	(308)
Mano, 1994	54	66.4	NA	Sweating dysfunction: 97%	Sweating dysfunction in PD reflects progressive involvement of the autonomic nervous system, with preganglionic or central pathway impairment seen in milder cases and postganglionic fiber involvement predominating in more advanced stages.	(314)
Sage, 1995	4	65	25%	Sweating dysfunction: 100%	Profuse sweating may reflect an off-period phenomenon in PD, triggered by subtherapeutic levodopa levels and reduced central dopaminergic stimulation.	(316)
Gubbay, 1966	2	67	0%	Hypothermia: 100%	PD may predispose to accidental hypothermia due to hypothalamic involvement and impaired thermoregulation.	(317)
Swinn, 2003	117	61.9	66%	Sweating dysfunction: 64%	Sweating disturbances are common in PD and are primarily linked to autonomic dysfunction, off periods, and dyskinesias.	(6)
Hashimoto, 2003	667	71.9	71%	Parkinsonism-hyperpyrexia	Malignant syndrome in PD may result from symptom deterioration alone but is more often triggered by rapid	(318)

				syndrome: 2.1%	withdrawal of dopaminergic therapy, dehydration, or infection, often accompanied by autonomic signs like diaphoresis.	
Saito, 2023	225	71.6	48.4%	Sweating dysfunction: 70.9%	Sudomotor abnormalities are common in PD and vary by motor subtype, with more severe dysfunction observed in akinetic-rigid and mixed forms, suggesting that sympathetic sudomotor involvement may be more closely associated with the pathophysiology of bradykinesia and rigidity than with tremor.	(319)

[a]Please see the main manuscript and reference list for the reference citations. NA, not available/not reported; PD, Parkinson's disease.

Table SXI. Clinical scales specifically designed for Parkinson’s disease.

Autonomic dysfunction	Scales	First author, year of publication	(Refs.)[a]
Gastrointestinal			
Sialorrhea	Sialorrhea Clinical Scale for Parkinson’s Disease (SCS-PD)	Perez Lloret, 2007	(421)
General	Gastrointestinal Dysfunction Scale for Parkinson’s Disease (GIDS-PD)	Camacho, 2021	(422)
Genitourinary			
Urinary	Questionnaire on Pelvic Organ Dysfunction in Parkinson’s Disease	Sakakibara, 2001	(423)
	Questionnaire on Bladder and Autonomic Dysfunction in Parkinson’s Disease	Pavy-Le, 2018	(424)
Global autonomic dysfunction	Scales for Outcomes in Parkinson’s Disease - Autonomic Dysfunction (SCOPA-AUT)	Visser, 2004	(86)
	Non-Motor Symptoms Questionnaire (NMSQuest)	Romenets, 2012	(72)
	Non-Motor Symptoms Scale for Parkinson's Disease (NMSS)	Chaudhuri, 2007	(425)
	Questionnaire on Autonomic dysfunction in Parkinson’s Disease	Pavy-Le, 2018	(424)
	Questionnaire on Symptoms of Autonomic Failure in Parkinson’s Disease	Pavy-Le, 2018	(424)
	MDS-Unified Parkinson's Disease Rating Scale (MDS-UPDRS) - Part I	Goetz, 2008	(426)
	Parkinson's Disease Questionnaire (PDQ-39)	Jenkinson, 1997	(427)
	Autonomic dysfunction in Parkinson’s disease	Goetz, 1986	(197)

[a]Please see the main manuscript and reference list for the reference citations.
